# Human papillomavirus (HPV) infection and prevalence of colorectal cancer: an updated systematic review and meta-analysis of global data

**DOI:** 10.1097/JS9.0000000000003426

**Published:** 2025-09-11

**Authors:** Mengqi Yang, Yujia Huo, Yedong Huang, Wenjun He, Qingyu Luo, Lin Zhang

**Affiliations:** a The Faculty of Pharmacy and Pharmaceutical Science, Monash University, Melbourne, Australia; b The School of Public Health and Preventive Medicine, Monash University, Melbourne, Australia; c Suzhou Industrial Park Monash Research Institute of Science and Technology, Monash University, Suzhou, China; d Monash University-Southeast University Joint Research Institute (Suzhou), Southeast University, Suzhou, China; e Clinical Oncology School of Fujian Medical University, Fujian Cancer Hospital, Fuzhou, Fujian, China; f Acacia Labs, SMU Institute for Global Health (SIGHT), Dermatology Hospital of Southern Mediscal University (SMU), Guangzhou, China; g Department of Medical Oncology, Dana-Farber Cancer Institute, Harvard Medical Schsool, Boston, MA, United States; h One Patient One Cure, Boston, MA, United States

**Keywords:** cancer epidemiology, colorectal cancer, HPV16, human papillomavirus, meta-analysis, systematic review, tumor virology

## Abstract

**Background:**

Human papillomavirus (HPV), a known oncogenic virus in cervical and anal cancers, has also been detected in colorectal tissues. However, evidence regarding its association with colorectal cancer (CRC) remains inconsistent. We conducted an updated systematic review and meta-analysis to clarify this relationship.

**Methods:**

Following PRISMA 2020 guidelines, we systematically searched PubMed, Embase, Web of Science, and Cochrane Library through May 2025 for observational studies (case–control and cross-sectional) assessing HPV prevalence in CRC patients versus controls. Data extraction was performed in duplicate. Pooled odds ratios (ORs) were estimated using random-effects models with logit transformation. Subgroup analyses and meta-regression examined the effects of geographic region, HPV genotype, detection method, and sample type. Risk of bias was assessed using the Newcastle–Ottawa Scale.

**Findings:**

Twenty case–control studies encompassing 1424 CRC cases and 1363 controls were included. The pooled OR for the association between HPV infection and CRC was 2.39 (95% CI: 1.69–3.09), with no significant heterogeneity (*I*^2^ = 0%). Associations were strongest in Asian studies (OR = 3.73) and in those using formalin-fixed paraffin-embedded (FFPE) tissues (OR = 3.56). Studies targeting HPV16 alone yielded higher effect sizes than those evaluating mixed or unspecified genotypes. Meta-regression confirmed region and genotype group as significant effect modifiers. Leave-one-out sensitivity analysis confirmed robustness. Egger’s test indicated marginal small-study bias (*P* = 0.074), but funnel plot symmetry suggested no serious publication bias.

**Conclusion:**

This meta-analysis confirms a significant link between HPV infection and colorectal cancer, suggesting HPV may play a broader oncogenic role beyond the anogenital tract. The findings highlight the need for genotype-aware, region-specific screening strategies, and support incorporating viral profiling into CRC prevention efforts, especially in underserved populations.

**HIGHLIGHTS**

- **HPV Infection is Significantly Associated with Colorectal Cancer Risk** This meta-analysis of 20 studies involving 1424 CRC cases and 1363 controls confirms that HPV infection is significantly associated with increased odds of CRC (pooled OR = 2.39, 95% CI: 1.69–3.09). The absence of statistical heterogeneity (*I*² = 0%) underscores the consistency and robustness of this association.
- **Subtype- and Region-Specific Patterns Reveal High-Risk Subgroups** Subgroup analyses reveal stronger associations in studies targeting HPV16 (OR = 3.78) and in Asian populations (OR = 3.73), highlighting biological and regional effect modifiers. These findings suggest that viral subtype profiling and population-specific screening strategies may enhance detection and prevention efforts.
- **Meta-Regression Strengthens Mechanistic Interpretation** Meta-regression confirms geographic region and genotype group as significant effect modifiers, suggesting potential roles for viral-host interactions and environmental co-factors. The findings support an evolving model of HPV as a molecular co-factor in colorectal carcinogenesis.
- **Implications for Virome-Informed Screening and Prevention** Results support the future development of virome-integrated screening tools for CRC, especially in high-risk populations such as MSM, immunocompromised individuals, or those with limited access to colonoscopy. These findings align with the WHO’s call for expanded early detection strategies in noncommunicable disease prevention.
- **Updated Evidence Base Enhances Clinical and Research Relevance** Compared to the team’s previous 2020 review, this updated analysis incorporates 8 new studies with enhanced detection techniques, mechanistic insights, and better geographic coverage – providing an evidence platform for precision public health approaches to CRC prevention.

## Introduction

Colorectal cancer (CRC) remains one of the most prevalent and lethal malignancies globally, with an estimated 2.1 million new cases and over 1 million deaths reported in 2024, according to the latest GLOBOCAN statistics^[1]^. While established risk factors, such as genetic predisposition, chronic inflammation, and lifestyle, are well documented, infectious agents are increasingly recognized as potential contributors to colorectal carcinogenesis^[2,3]^. Among them, human papillomavirus (HPV), a double-stranded DNA virus with established oncogenicity in cervical, anal, and oropharyngeal cancers, has been recurrently detected in colorectal tumors^[4,5]^.

This detection has led to persistent debate regarding the potential role of HPV in colorectal tumorigenesis. Several case–control studies have reported significantly elevated CRC risk in HPV-positive individuals^[6,7]^, while others have found no such association^[8,9]^. Methodological heterogeneity – such as differences in HPV genotyping protocols, tissue source (biopsy vs. fresh-frozen), and regional prevalence – may underlie these inconsistencies^[10,11]^. To address this uncertainty, our group conducted a global systematic review and meta-analysis in 2020, which suggested a pooled association between HPV infection and CRC risk^[12]^. A follow-up analysis in 2022 reaffirmed this association, reporting a sixfold increased risk among HPV-positive patients^[13]^.

Over the past 5 years, the evidence base has expanded substantially. Our updated search identified eight eligible studies published between 2020 and 2025, offering novel insights into HPV detection, regional patterns, and co-infection dynamics^[6,7,14–19]^. Additionally, newer studies incorporate mechanistic hypotheses involving viral integration, host–microbiota interactions, and viral co-infections such as Epstein–Barr virus (EBV), providing critical context for previously unexplained epidemiological heterogeneity^[16,20–22]^. Nevertheless, prior meta-analyses, including our own 2020 review, did not systematically compare HPV prevalence across different sample processing types (e.g., FFPE vs fresh/preserved tissues) or quantify genotype-specific associations for HPV16, HPV18, and other high-risk types^[12,23]^.

Mechanistically, high-risk HPV types, especially HPV-16 and HPV-18, can integrate into host genomes and disrupt tumor suppressor pathways, thereby promoting cellular transformation^[16,22,24]^. Transcriptionally active HPV has been detected in colorectal tumor tissues, indicating viral gene expression rather than mere DNA presence^[16,22]^. Co-infection with Epstein–Barr virus (EBV) may further contribute to inflammation-mediated oncogenesis through immune evasion and chronic epithelial disruption^[21,25]^. Emerging evidence also suggests that gut dysbiosis and inflammation can facilitate HPV persistence, viral integration, and immune escape, particularly in the anorectal region^[26–28]^.

From a public health standpoint, the inclusion of HPV screening strategies in CRC prevention programs programs is gaining interest, particularly for underserved or high-risk populations^[13,29]^. The World Health Organization’s 2023–2030 roadmap for noncommunicable disease control emphasizes strengthening cancer prevention, including early detection and screening for high-burden cancers^[30]^. Multi-pathogen self-sampling and non-invasive molecular screening may be particularly beneficial in populations with low colonoscopy uptake, such as men who have sex with men (MSM), immunocompromised individuals, or those in low-resource settings^[31,32]^.

Building on the expanding literature and emerging mechanistic hypotheses, we conducted an updated systematic review and meta-analysis to reassess the relationship between HPV infection and colorectal cancer. By incorporating new studies published through May 2025, we aimed to refine the pooled effect estimate. Specific unresolved questions from prior reviews were also examined, particularly whether HPV prevalence differs by sample processing type (e.g., FFPE vs fresh/preserved tissues) and whether genotype-specific risks vary across HPV16, HPV18, and other high-risk types. We further investigated sources of heterogeneity, including region and detection method, and interpreted the findings within the context of recent insights on viral co-infections and microenvironmental influences. This work seeks to clarify the epidemiological signal and inform futuse screening and prevention strategies that integrate virome-based risk stratification.

## Methods

### Protocol and reporting framework

This systematic review and meta-analysis was conducted in accordance with the PRISMA 2020 guidelines^[33]^. The study protocol was prospectively registered on the PROSPERO International Prospective Register of Systematic Reviews, ensuring methodological transparency and reproducibility. This review serves as an updated extension of our previously published meta-analysis on the same topic, with an identical search strategy and statistical approach but an expanded literature timeframe to include studies published through May 2025. The study aimed to assess the association between HPV infection and the risk of CRC. This systematic review was conducted and reported in accordance with the AMSTAR 2 (A Measurement Tool to Assess Systematic Reviews) guidelines^[34]^.

### AI transparency statement

This review was also conducted and reported in accordance with the TITAN 2025 Guidelines for transparency and integrity in AI-assisted scientific reporting^[35]^. The corresponding checklists are provided as Supplemental Digital Content, Materials, http://links.lww.com/JS9/F144 No artificial intelligence (AI) tools were used in any stage of the study, including study design, data analysis, or manuscript drafting.

### Search strategy and eligibility criteria

A comprehensive literature search was performed in PubMed, Embase, Web of Science, and the Cochrane Library, covering all records from database inception to 1 May 2025. The search strategy combined Medical Subject Headings (MeSH) and free-text terms related to “human papillomavirus,” “colorectal cancer,” “colorectal neoplasms,” and their variants. Full search algorithms for each database are provided in Supplementary Digital Content, Table S1, available at: http://links.lww.com/JS9/F46

To ensure a comprehensive and cumulative evidence base, we extended the original search window to include new studies published between January 2020 and May 2025, and integrated them with the dataset from our prior review. All eligible studies were re-analyzed using the same eligibility criteria and statistical approach to ensure consistency across time points.

Studies were included if they met the following criteria: (1) observational design (case-control, cross-sectional, or cohort); (2) histologically confirmed CRC; (3) HPV detection through molecular techniques such as PCR, hybrid capture, or *in situ* hybridization; and (4) availability of sufficient data to compute odds ratios (ORs) with 95% confidence intervals (CIs). Studies were excluded if they lacked a control group, reported zero events in both comparison arms, or were reviews, commentaries, or duplicates.

### Data extraction and quality assessment

Two reviewers independently screened all titles, abstracts, and full texts. Data extraction was performed using a pre-piloted Excel-based template designed for this study. To ensure accuracy and internal consistency, double data entry and independent cross-checking were conducted. Any discrepancies were resolved by discussion or, if needed, adjudication by a third reviewer. Extracted variables included: first author, publication year, country, study design, sample type (e.g., fresh tissue, FFPE, or stool), HPV detection method, HPV genotypes tested, and numbers of HPV-positive and -negative individuals in both case and control groups.

Risk of bias for included observational studies was assessed using the Newcastle–Ottawa Scale (NOS), which evaluates participant selection, comparability, and outcome/exposure ascertainment^[36]^. Two reviewers independently scored each study, and discrepancies were resolved via consensus. Studies scoring ≥6 were considered moderate to high quality. In addition, the Grading of Recommendations, Assessment, Development and Evaluations (GRADE) framework was applied to evaluate the certainty of evidence for the primary outcome (i.e., HPV infection and CRC association). The assessment considered risk of bias, inconsistency, indirectness, imprecision, and publication bias across studies. Information on blinding of laboratory personnel and the use of confirmatory genotyping was planned for extraction where reported in the original articles. For observational designs such as case–control studies, these details are often not explicitly described; therefore, they were noted when available and considered in the quality assessment process.

### Statistical analysis

Random-effects models using the DerSimonian and Laird method were applied to compute pooled ORs and 95% CIs, accounting for potential between-study variability^[37]^. ORs were logit-transformed prior to pooling to stabilize variance. Heterogeneity was assessed using the *I*^2^ statistic and Cochran’s Q test, with *I*^2^ > 50% considered indicative of substantial heterogeneity^[38,39]^.

Prespecified subgroup analyses were conducted by geographic region (Asia vs. non-Asia), HPV genotype category (HPV16 only, HPV16 + 18, high-risk mixed), detection method (PCR vs. non-PCR), and sample type (FFPE tissue, fresh tissue, or stool). These subgroup variables were pre-specified based on biological plausibility and prior literature. Sample type categories were defined a priori as follows: FFPE referred to specimens processed by formalin fixation and paraffin embedding; fresh tissue included surgical samples or tissues preserved by non-paraffin methods such as snap-freezing or stabilizing media; and stool samples referred to fecal specimens tested for HPV DNA. For studies reporting multiple specimen types, the predominant type used for HPV detection was selected to ensure mutually exclusive subgroup assignment. Sensitivity analyses using leave-one-out procedures were performed to evaluate the influence of individual studies on the overall effect estimates.

### Assessment of heterogeneity and publication bias

Univariable meta-regression analyses were conducted to explore potential sources of heterogeneity using publication year, geographic region, detection method, and genotype group as covariates. Bubble plots were generated to visualize covariate effects^[40]^. We reported both the adjusted *R*^2^ (proportion of between-study variance explained by the model) and residual heterogeneity (τ^2^) for all meta-regression models. To assess multicollinearity between covariates in multivariable models, we calculated variance inflation factors (VIF), with values >5 indicating potential collinearity.

Publication bias was evaluated using funnel plot symmetry and Egger’s linear regression test^[41,42]^, with *P* < 0.10 indicating potential small-study effects. All statistical analyses were performed using Stata version 18.5 (StataCorp LLC, College Station, TX, USA).

## Results

### Study selection and characteristics

**Figure 1.**
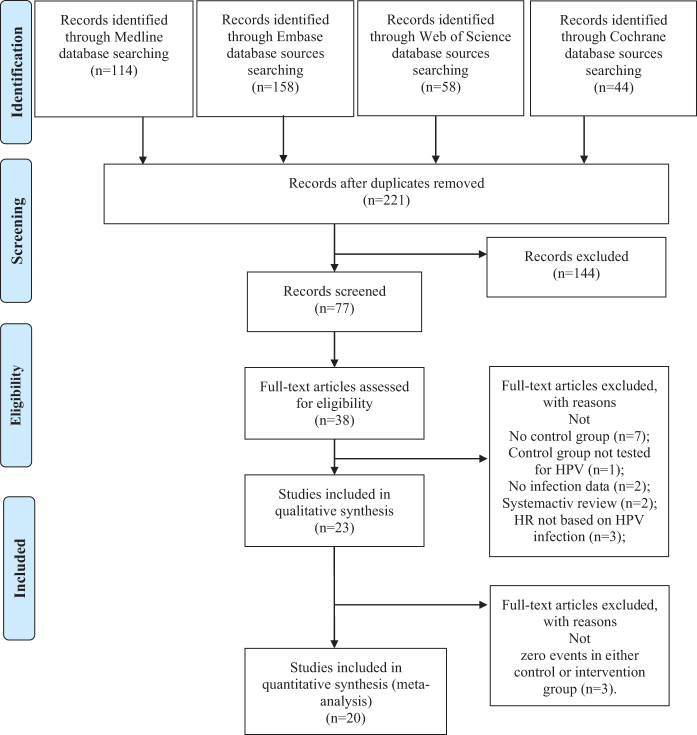
PRISMA 2020 flow diagram for the systematic review of HPV infection and colorectal cancer.

A total of 374 records were identified from four major databases – PubMed (*n* = 114), Embase (*n* = 158), Web of Science (*n* = 58), and the Cochrane Library (*n* = 44). After removing duplicates, 221 unique records remained for title and abstract screening. Of these, 144 were excluded based on relevance. Thirty-eight full-text articles were assessed for eligibility, and 15 were further excluded due to lack of a control group (*n* = 7), no HPV testing in controls (*n* = 1), missing infection data (*n* = 2), non-original articles (*n* = 2), or outcomes unrelated to HPV infection (*n* = 3). Ultimately, 23 observational studies were included in the qualitative synthesis, of which 20 met the criteria for quantitative analysis after excluding studies with zero events in both arms (Fig. 1).

These 20 studies included a combined total of 1424 colorectal cancer cases and 1363 control subjects. Studies were conducted across diverse regions: nine from Asia, six from Europe, and five from the Americas. Sample types varied, with six studies using formalin-fixed paraffin-embedded (FFPE) tissues, nine using fresh or preserved samples, and five analyzing paired tumor and adjacent normal tissues. Conventional PCR was the predominant detection method (*n* = 11), followed by nested PCR (*n* = 6) and hybrid methods (*n* = 3). Genotypic characterization was inconsistent: four studies focused on HPV16 alone, two assessed HPV16 and 18, six evaluated multiple high-risk types, and eight did not report specific genotypes. A detailed summary of included studies is presented in Table 1.

**Table 1:** Characteristics of included studies assessing the association between HPV infection and colorectal cancer

First Author	Year	Country	Research design	Pathology Sample	Detection method	Primer	HPV genotype	Cases	Case (HPV +)	Controls	Control (HPV +)	OR	LCI	UCI
Bodaghi, S.^[43]^	2005	USA	Case–control	Tumor and tumor-adjacent	Nested-PCR	PGMY09/11 + GP5 +/6 +	High-risk mixed	55	23	52	15	1.8	0.8	4
Buyru, N.^[44]^	2006	Istanbul	Case–control	Tissue	Conventional PCR	MY09/11	Low + high risk	53	43	53	10	4.3	1.87	10.53
Damin, D. C.^[45]^	2007	Brazil	Case–control	Tissues	Nested-PCR	MY09/11 + GP5 +/6 + + E6/E7	Low + high risk	72	46	72	37	1.7	0.9	3.3
Laskar, R. S.^[46]^	2015	India	Case–control	Fresh tumor + normal tissue	Conventional PCR	MY09/11 + GP5 +/6 +	16 + 18	93	34	30	6	1.82	0.66	5.83
Lee, Y. M.^[47]^	2001	China	Case–control	Tissue	Conventional PCR	HPV-18 primers	18 only	19	16	19	10	4.8	1	22.1
Liu, F.^[48]^	2011	China	Case–control	Fresh tumor + distant tissue + blood	Nested-PCR	PGMY09/11 + GP5 +/6 +	Low + high risk	96	28	96	3	12.7	3.7	67.4
Picanco-Junior, O. M.^[49]^	2014	Brazil	Case–control	FFPE	PCR + Dot blot	E7 only	Low + high risk	79	36	65	5	5.92	2.12	20.29
Salepci, T.^[50]^	2009	Turkey	Case–control	Tumor + normal tissues	Conventional PCR	MY09/11	Low + high risk	56	46	56	18	2.55	1.26	5.26
Tanzi, E.^[51]^	2015	Italy	Case–control	FFPE + adjacent normal tissue	Nested PCR	ELSI-f/r + GP5 +/6 +	Low + high risk	57	9	57	5	1.8	0.5	7.24
Vuitton, L.^[52]^	2017	France	Case–control	FFPE	PCR + INNO-LiPA	Not specified	16 + 18	257	9	210	4	1.83	0.5	8.28
Yu, H. G.^[53]^	2002	China	Case–control	Tumor + adjacent tissue	Conventional PCR	Not specified	16 + 18	32	7	32	1	7	0.8	325.08
Zhang, J. C.^[54]^	2012	China	Case–control	Fresh tumor + adjacent tissue	Conventional PCR	E7	16 only	106	48	106	7	6.86	2.89	18.67
Tavakolian^[14]^	2020	Iran	Case–control	FFPE	Conventional PCR	GP5 +/6 +	16 only	25	8	25	1	10.67	1.21	94.04
Galati^[18]^	2023	Italy	Case–control	FFPE	Conventional PCR	Not rep.	16 only	134	51	134	20	3.51	1.91	6.44
Ambrosio^[17]^	2023	Brazil	Case–control	FFPE	Conventional PCR	Not rep.	Not reported	74	14	22	0	7	1.5	32
Abedi Elkhichi^[19]^	2024	Iran	Case–control	FFPE	Conventional PCR	Not rep.	Not reported	60	15	60	1	19.67	2.46	157.2
Hsu^[15]^	2022	Taiwan	Case–control	Likely FFPE (NR)	Conventional PCR	Not rep.	Not reported	131	49	131	9	8.43	3.87	18.36
Kadhem Mallakh^[16]^	2022	Iraq	Case–control	FFPE	Conventional PCR	Not rep.	Not reported	40	22	20	2	11	2.22	54.47
Niya^[6]^	2022	Iran	Case–control	FFPE	Conventional PCR	Not rep.	16 + 18	80	31	80	10	4.33	1.96	9.57
Pan^[7]^	2024	China	Case vs. adjacent	FFPE	Conventional PCR	SPF10	Low + high risk	86	41	86	8	7.11	3	16.86

Note: FFPE = formalin-fixed paraffin-embedded; PCR = polymerase chain reaction; INNO-LiPA = line probe assay; OR = odds ratio; LCI = lower confidence interval; UCI = upper confidence interval; HPV = human papillomavirus; E6/E7 = early region oncogenes E6 and E7 of HPV; PGMY09/11, GP5 +/6 +, MY09/11, ELSI-f/r = primer sets used for HPV DNA amplification. “Low + high risk” refers to studies that tested both low-risk (e.g., HPV 6/11) and high-risk (e.g., HPV 16/18) genotypes. “High-risk mixed” refers to studies that tested multiple high-risk types only. “Not specified” indicates that the genotype or primer details were not reported in the original study.

### Study quality and evidence certainty

Of the 20 studies included in the meta-analysis, 17 were rated as high quality (NOS score ≥7), and 3 as moderate quality (scores 5–6), with no study falling into the low-quality category. The most common limitations were limited control for confounders and incomplete reporting of exposure ascertainment.

The certainty of evidence for each key outcome was further evaluated using the GRADE framework. The overall association between HPV infection and CRC was rated as moderate-certainty evidence, supported by consistent findings across regions, negligible heterogeneity (*I*^2^ = 0%), and precise pooled estimates. Subgroup findings – such as the effect observed in HPV16-only studies – were rated as low certainty, while the FFPE-based subgroup was rated as moderate, primarily due to imprecision from small sample sizes and wide confidence intervals.

These quality and certainty assessments lend methodological confidence to the robustness of our pooled estimates and subgroup interpretations, while also identifying areas – such as genotype-specific associations – that require further evidence for stronger causal inference. Detailed assessments are provided in Supplementary Digital Content, Table S1, available at: http://links.lww.com/JS9/F46 (NOS) and Supplementary Digital Content, Table S2, available at: http://links.lww.com/JS9/F47 *(GRADE)*.

### Overall pooled association

Across the 20 studies included in the quantitative synthesis, the pooled OR for the association between HPV infection and colorectal cancer was 2.39 (95% CI: 1.69–3.09), based on a random-effects model. The results showed a statistically relevant increase in CRC risk among HPV-infected individuals.

**Figure 2.**
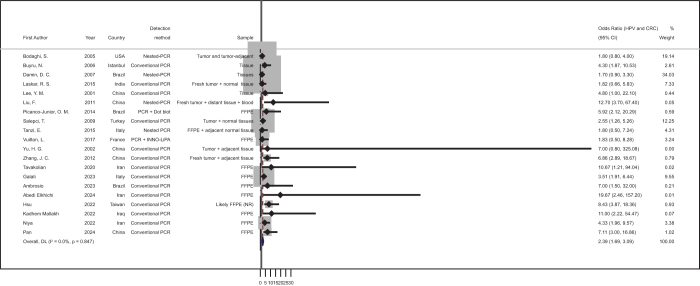
Forest plot of the overall association between HPV infection and colorectal cancer. Each horizontal line represents a study-specific OR with 95% CI for the association between HPV infection and CRC. The pooled OR was calculated using a random-effects model. Study weights are proportional to inverse-variance and indicated on the right.

The forest plot (Fig. 2) revealed that the majority of individual studies reported a positive association, with 15 of 20 studies yielding ORs above 1. Studies contributing the greatest weight to the pooled estimate were those with larger sample sizes and narrower confidence intervals. In contrast, studies with extreme ORs and wide CIs, most notably Yu et al. (OR = 7.00; 95% CI: 0.80–325.08), contributed minimal weight (<0.01%) and had no material influence on the overall result.

No significant statistical heterogeneity was observed. The *I*^2^ value was 0.0%, with Cochran’s Q = 12.84 (df = 19, *P* = 0.847) and between-study variance (τ^2^) estimated at zero. These metrics indicate high consistency across studies and justify the application of a pooled estimate in further exploratory analyses.

### Subgroup analyses

Prespecified subgroup analyses were performed to explore the impact of methodological and geographical variables on the pooled effect estimate. When stratified by specimen type, studies utilizing formalin-fixed paraffin-embedded (FFPE) samples demonstrated the strongest association between HPV infection and colorectal cancer (pooled OR = 3.56, 95% CI: 2.11–5.01)^[6,7,14–19,51,52,55]^, as shown in Supplementary Digital Content, Figure 1, Available at: http://links.lww.com/JS9/F45 Studies using fresh or preserved tissue yielded a lower, non-significant pooled OR of 1.92 (95% CI: 0.77–3.07)^[44,45,47]^. A subset of studies examined paired tumor-adjacent tissues reported an intermediate effect (OR = 2.09, 95% CI: 0.84–3.34)^[43,50,53]^. Although FFPE-based studies tended to yield higher estimates, the difference across specimen types was not statistically significant (Q = 3.37, df = 2, *P* = 0.185), as shown in Supplementary Digital Content, Figure 1, available at: http://links.lww.com/JS9/F45

Regional variation was also explored (Supplementary Digital Content, Figure 2, available at: http://links.lww.com/JS9/F45). The pooled OR for Asian studies was 3.73 (95% CI: 1.87–5.60)^[6,7,14–16,19,46–48,53],^ notably higher than estimates from European (2.67, 95% CI: 1.38–3.97)^[18,50–52]^, and American (1.80, 95% CI: 0.85–2.76) studies^[17,43,45,55]^. These between-region differences were also not statistically significant (Q = 3.62, df = 2, *P* = 0.164), though the observed pattern may reflect underlying differences in genotype distribution, population immunity, or other contextual factors.

**Figure 3.**
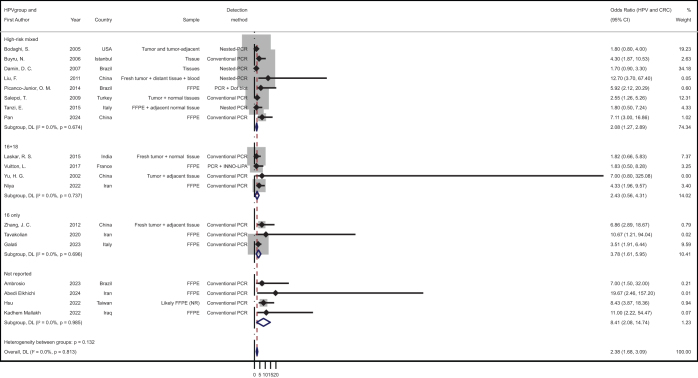
Subgroup analysis of the association between HPV infection and CRC by HPV genotype. The forest plot presents stratified effect estimates based on HPV genotype categories, including high-risk mixed types, HPV16 only, HPV16 + 18, and studies without genotype specification. Subgrouping was based on reported PCR primer targets and genotyping scope. Estimates were calculated using a random-effects model.

Subgrouping by HPV genotype revealed additional variation, shown in the Figure 3. Studies limited to HPV16 yielded the highest pooled OR of 3.78 (95% CI: 1.61–5.96)^[14,18,54]^, followed by studies detecting HPV16 + 18 (2.43, 95% CI: 0.56–4.31) and studies assessing multiple high-risk types (2.08, 95% CI: 1.27–2.89). Studies without genotype specification still produced an elevated pooled OR of 8.41 (95% CI: 2.08–14.74), though with a wide confidence interval^[15–17,19]^. The between-subgroup difference was not statistically significant (Q = 5.61, df = 3, *P* = 0.132), but results remained directionally consistent across categories.

### Sensitivity analysis

To evaluate the stability of the pooled estimate, a leave-one-out sensitivity analysis was conducted. Removal of any individual study from the meta-analysis did not materially alter the overall effect size. The pooled ORs obtained across iterations ranged narrowly from 2.31 to 2.47, and all confidence intervals remained statistically significant (i.e., not crossing 1.0). The leave-one-out plot (Supplementary Digital Content, Figure 3, available at: http://links.lww.com/JS9/F45) showed no outlier exerting disproportionate influence on the summary estimate. Notably, exclusion of the Yu et al study^[53]^, which reported an extreme effect size and wide uncertainty, did not affect the direction or significance of the overall result. These findings affirm the numerical and statistical robustness of the pooled association.

### Meta-regression

Meta-regression analyses were conducted to assess whether study-level covariates accounted for heterogeneity in effect sizes. In multivariable models, studies from Asia reported significantly higher log-transformed odds ratios compared to other regions (coefficient = 0.89, SE = 0.32, *P* = 0.015), with an adjusted *R*^2^ of 100.0% and no residual heterogeneity (τ^2^ = 0.000)^[6,7,14–16,19,43–45,50]^.

In contrast, studies detecting both HPV16 and 18 reported significantly lower effect sizes than those without genotype reporting (coefficient = −0.98, SE = 0.42, *P* = 0.036)^[6,46,52,53]^. Detection method (nested vs. conventional PCR) was not significantly associated with effect size (*P* = 0.203). In the multivariable model incorporating region, detection method, and genotype group, the model reached overall significance (F = 3.78, df = 6,13; *P* = 0.0211), with an adjusted *R*^2^ of 100%. The regional variable remained statistically significant (*P* = 0.022), while detection method and genotype group did not retain significance (*P* = 0.158 and 0.091, respectively). Bubble plots of the univariable regressions are presented in Supplementary Digital Content, Figure 4&5, available at: http://links.lww.com/JS9/F45. To explore potential interactions between key moderators, we conducted meta-interaction analyses by including interaction terms (e.g., sample type × genotype) in meta-regression models. Significance was assessed at *P* < 0.05. While no interaction reached statistical significance, a trend toward synergistic effects was observed for FFPE sample use combined with HPV16 detection. No significant interaction was detected between FFPE sample type and HPV16 detection (*P* = 0.214), although the direction of effect was suggestive of potential attenuation.

### Publication bias

Visual inspection of the funnel plot (Supplementary Digital Content, Figure 6, available at: http://links.lww.com/JS9/F45) revealed a generally symmetric distribution of studies around the pooled effect estimate. Larger studies with smaller standard errors clustered near the top of the plot, while smaller studies were more widely scattered, as expected. No marked asymmetry or directional clustering was observed. Egger’s regression test yielded a bias intercept of 1.40 (*P* = 0.074), suggesting a marginal small-study effect. However, the visual symmetry of the funnel plot and consistency across study sizes argue against the presence of substantial publication bias.

## Discussion

This updated meta-analysis provides compelling evidence for a consistent and statistically significant association between HPV infection and CRC, with a pooled OR was 2.39 and no observed heterogeneity (*I*^2^ = 0%). The consistency of this effect across diverse study populations and detection methods strengthens the epidemiological credibility of the finding. Subgroup and meta-regression analyses further identified region, HPV genotype, and sample type as potential effect modifiers, with geographic region emerging as the most consistent source of variation. These results underscore the need to consider contextual and biological factors when interpreting the strength of the HPV–CRC association across different settings.

These quality and certainty assessments lend methodological confidence to the robustness of our pooled estimates and subgroup interpretations. Building on these data, we conducted a comparative synthesis with our 2020 meta-analysis to assess the stability and added value of the updated evidence. The original study reported a pooled OR of 2.12^[12]^. The present analysis expands the evidence base by over 40% in sample size and integrates methodological advances such as nested PCR, INNO-LiPA genotyping, and co-infection screening. It identifies HPV16-specific effects, clarifies regional variation, and reveals context-dependent amplification of the HPV–CRC signal – shifting the association from a broad epidemiological link toward a more stratified, mechanistically informed framework.These enhancements reinforce the credibility of the observed relationship and broaden its translational relevance for precision screening and risk stratification.

Of particular note is the HPV16 subtype, which exhibited a notably higher effect size than broader or mixed-genotype categories. This finding aligns with its known oncogenic profile, characterized by a high propensity for host genome integration and sustained expression of E6 and E7 oncoproteins^[24,56,57]^. In contrast, high-risk HPV18 has shown less consistent associations in colorectal tissue, possibly reflecting tissue tropism differences or lower prevalence in the lower gastrointestinal tract^[43]^. The stronger signal observed in FFPE specimens may be partly attributable to enhanced preservation of viral DNA, particularly integrated genomes, and improved detection sensitivity in PCR-based assays^[58,59]^. However, residual confounding from laboratory protocols or sample processing cannot be excluded, and future studies should consider matching detection methods across sample types^[60].^ Furthermore, while no synergistic interaction was observed between FFPE and HPV16 detection, the signal directionality may warrant further exploration in larger datasets.

In this mechanistic context, accumulating molecular data are consistent with HPV potentially acting as a co-factor in colorectal carcinogenesis, although a causal role remains unconfirmed. Multiple studies included in this review detected transcriptionally active E6/E7 mRNA, implicating HPV in disrupting p53 and Rb pathways^[16,22,24,61]^. HPV16 has also been linked to p16INK4a overexpression in CRC tissues, mirroring patterns established in cervical, anal, and oropharyngeal cancers^[62]^. Co-detection of EBV in HPV-positive colorectal samples may indicate viral co-occurrence^[63]^. While EBV could theoretically promote persistent HPV infection via immunosuppressive cytokines such as IL-10 and TGF-β, these mechanisms remain speculative in CRC and warrant targeted investigation^[21,25,64]^.

Similarly, HPV-related oncogenesis may be shaped by microbiome composition. In HPV-positive CRC, enrichment of *Fusobacterium nucleatum* and depletion of *Bifidobacterium* have been observed^[9,17,65,66]^. *Fusobacterium nucleatum* can modulate epithelial-to-mesenchymal transition, immune evasion, and β-catenin signaling, potentially affecting viral persistence.^[65,67]^. These host–virus–microbiota interactions have been hypothesized to modulate susceptibility, but alternative explanations such as shared environmental exposures cannot be excluded.

Region-specific heterogeneity emerged as a consistent moderator, with Asian studies reporting significantly higher effect sizes than their Western counterparts^[68,69]^. Such differences may be hypothesized to relate to underlying variation in host genetics (e.g., HLA polymorphisms), dietary patterns, sanitation, or exposure to enteric pathogens, but causal mechanisms remain to be clarified and require further investigation^[70]^. From a screening equity perspective, this variation underscores the importance of context-specific strategies, particularly in high-burden or resource-limited settings^[70,71]^. To move beyond association and toward causal inference, future research should prioritize longitudinal, multi-center cohort studies. Key populations include adults under 50 years, men who have sex with men (MSM), and immunocompromised individuals, who are disproportionately affected by persistent HPV infection and remain underrepresented in CRC prevention programs^[15,68]^. Prospective tracking of HPV status, immune markers, and microbiota composition – via stool, rectal swabs, and blood collected every 6–12 months – would enable detailed assessment of infection dynamics and oncogenic progression. Target endpoints should include adenoma formation, histologic dysplasia, and viral integration events. Complementary Mendelian randomization approaches, focusing on genetic variants involved in viral entry (e.g., syndecan-1, integrin α6) or immune surveillance, may also help disentangle causal from correlative associations^[15,68]^.

Clinically, these updated findings support the consideration of HPV-based CRC risk stratification in specific populations. If future longitudinal studies establish a causal or clinically actionable link, multi-target stool assays integrating HPV detection could potentially help refine screening algorithms for FIT-positive and colonoscopy-negative individuals^[68,72]^. Although no stool- or anal-based HPV self-sampling kits have been validated for CRC screening, pilot studies in MSM and HIV-positive populations demonstrate the acceptability and diagnostic adequacy of self-collected anal swabs^[32,73]^. Drawing from the cervical cancer screening experience, where self-sampling has proven both cost-effective and impactful in underserved populations^[74]^, the adaptation of these tools to CRC prevention contexts represents a promising, though still nascent, direction. Future implementation studies are needed to validate the diagnostic accuracy, user acceptability, and health system integration of HPV-based self-sampling strategies for CRC screening. Although no stool- or anal-based HPV self-sampling method has yet been validated for CRC, evidence from cervical cancer programs offers a strong translational precedent. Randomized trials and implementation studies have shown that self-collection increases screening uptake and yields diagnostically adequate samples across diverse populations^[31,73,74]^. These findings support the feasibility of adapting similar approaches for CRC prevention, especially in high-risk or under-screened groups such as MSM^[68,75]^. However, the integration of HPV-based stool assays into routine CRC screening remains technically and economically challenging. No commercialized kits are currently available, and several barriers persist – including DNA stability in fecal samples, compatibility with existing FIT or fecal DNA tests, quality control of viral detection, and cost-effectiveness across healthcare settings. Until these challenges are addressed, stool-based virome screening should be regarded as an investigational adjunct rather than a replacement for established CRC screening modalities.

Several methodological and contextual limitations should be considered when interpreting our findings. First, all included studies were observational in nature – predominantly case–control in design—which precludes causal inference and may introduce selection bias (as most were hospital-based) and residual confounding. Second, there was heterogeneity in HPV detection methods, including differences in sample types (FFPE vs fresh tissue; tumor vs control), DNA/RNA extraction protocols, primer sets, and detection platforms (PCR, qPCR, ISH), as well as varying threshold criteria. Some studies used p16 as a surrogate marker, which may not directly reflect HPV DNA or RNA status. Third, HPV genotyping lacked standardization across studies, with variable detection panels, algorithms, and reporting practices. Few studies evaluated viral integration status or transcriptional activity (e.g., E6/E7 mRNA). Fourth, potential confounding by co-infections and regional factors was rarely addressed, including EBV or other oncogenic viruses, as well as variation in background HPV prevalence, genotype distribution, sexual behavior patterns, vaccine coverage, and laboratory capacity. Finally, differences in control tissue selection and the extent of covariate adjustment (e.g., age, sex, tumor site, stage, MSI status) further limited comparability across studies.

Looking ahead, the integration of virome data, including HPV subtype profiling, transcriptional activity, and co-infection patterns (e.g., Epstein–Barr virus), with mucosal immune and microbial biomarkers will be key to advancing from associative findings to biologically grounded prevention strategies. One key avenue is the development of multi-analyte, stool-based assays that combine HPV and EBV detection with microbial dysbiosis signatures, such as *Fusobacterium nucleatum* enrichment, to capture potential host–virus–microbiota interactions. These platforms could serve as minimally invasive precision tools for identifying high-risk individuals – particularly those who test positive on fecal immunochemical tests (FIT) but are unable or unwilling to undergo colonoscopy^[76–78]^. Examples such as Cologuard® and emerging cfDNA-based diagnostics illustrate the feasibility of integrating viral and microbial biomarkers into early CRC detection strategies^[79,80]^.

To advance mechanistic understanding, we further propose the establishment of longitudinal multi-site cohort studies. These would enroll high-risk groups, such as MSM, HIV-positive individuals, or immunosuppressed patients, with serial collection of stool, rectal swabs, and blood every 6–12 months. Key endpoints should include persistent HPV/EBV infection, relevant cytokine alterations (e.g., IL-10, TGF-β), and the development of colorectal precursors such as adenomas or dysplasia^[15,17,25].^ Combined with advanced molecular assays (e.g., E6/E7 mRNA expression, metagenomics), such studies could illuminate the causal pathways linking viral colonization to malignant transformation. Complementary Mendelian randomization analyses, focusing on genes involved in viral entry or immune modulation (e.g., *SDC1, ITGA6*), could further disentangle association from causation^[21,64]^.

As colorectal cancer screening continues to evolve toward precision public health, these integrated, etiology-driven approaches hold substantial promise for earlier detection, individualized risk stratification, and closing equity gaps in cancer prevention.

## Conclusion

In summary, this updated meta-analysis confirms a statistically significant and robust association between HPV infection and CRC, with consistent findings across studies and negligible heterogeneity. Subgroup and meta-regression analyses suggest that regional, genotypic, and methodological factors may modify the observed effect, with particularly strong associations in Asian populations and HPV16-targeted studies. While causality cannot yet be inferred, the convergence of epidemiological patterns and molecular plausibility supports a potential role for HPV as a co-factor in colorectal carcinogenesis, especially in the context of viral co-infections and gut dysbiosis. These findings underscore the promise of virome-informed, non-invasive screening strategies for high-risk or underserved populations, such as MSM and immunocompromised individuals. Moving forward, longitudinal cohort studies and multi-analyte diagnostic platforms will be critical to clarifying causal pathways and informing equitable, biologically driven CRC prevention strategies.

## Supplementary Material

## Data Availability

Data sharing is not applicable as no data sets were generated and/or analysed for this study.

## Research registration unique identifying number (UIN)

This systematic review was prospectively registered with the International Prospective Register of Systematic Reviews (PROSPERO). No amendments were made to the original protocol. Any changes arising during the peer-review process will be transparently documented in the final publication.
