# Supplementary material for: Human papillomavirus (HPV) infection and prevalence of colorectal cancer: an updated systematic review and meta-analysis of global data
Source: Int J Surg. 2025 Sep 11;112(1):1815–25. doi: 10.1097/JS9.0000000000003426 (PMC12825822; doi:10.1097/JS9.0000000000003426)
Supplement: Supplementary file 1 [file js9-112-1815-001.docx]

| **TITAN Guideline Checklist 2025** | | | |
| --- | --- | --- | --- |
| **Topic** | **Item** | **Description** | **Page number** |
| **Artificial Intelligence (AI) (some journals may prefer this in the methods and/or acknowledgments section and it should also be declared in the cover letter)** | 1 | **Declaration of whether any AI was used in the research and manuscript development**  **No AI was used in the research or manuscript development.** |  |
|  | 1a | **Purpose and Scope of AI Use** |  |
|  | 1b | **AI Tool(s) and Configuration** |  |
|  | 1c | **Data Inputs and Safeguards** |  |
|  | 1d | **Human Oversight and Verification** |  |
|  | 1e | **Bias, Ethics and Regulatory Compliance** |  |
|  | 1f | **Reproducibility and Transparency** |  |
