# Supplementary material for: Human papillomavirus (HPV) infection and prevalence of colorectal cancer: an updated systematic review and meta-analysis of global data
Source: Int J Surg. 2025 Sep 11;112(1):1815–25. doi: 10.1097/JS9.0000000000003426 (PMC12825822; doi:10.1097/JS9.0000000000003426)
Supplement: Supplementary file 2 [file js9-112-1815-002.docx]

# Supplementary Table 1. GRADE Evidence Profile for Key Outcomes

| Outcome | Study design | Risk of bias | Inconsistency | Indirectness | Imprecision | Publication bias | Overall certainty |
| --- | --- | --- | --- | --- | --- | --- | --- |
| Overall association between HPV and CRC | Observational (20 studies) | Not serious | Not serious (I² = 0%) | Not serious | Not serious (CI does not cross null) | No serious bias (Egger’s p = 0.074) | Moderate |
| Association in FFPE vs fresh tissue | Observational (15 studies) | Not serious | Not serious (I² = 0%) | Not serious | Moderate concern (fresh tissue subgroup imprecise) | No serious bias | Moderate |
| Association in HPV16-only studies | Observational (4 studies) | Serious (small N, some NOS <6) | Not serious | Not serious | Serious (wide CI) | Unclear (few studies) | Low |
| Association in Asia vs non-Asia | Observational (20 studies) | Not serious | Not serious (I² = 0%) | Not serious | Minor concern (America subgroup imprecise) | No serious bias | Moderate |
