# Supplementary material for: Human papillomavirus (HPV) infection and prevalence of colorectal cancer: an updated systematic review and meta-analysis of global data
Source: Int J Surg. 2025 Sep 11;112(1):1815–25. doi: 10.1097/JS9.0000000000003426 (PMC12825822; doi:10.1097/JS9.0000000000003426)
Supplement: Supplementary file 3 [file js9-112-1815-003.docx]

# Supplementary Table 2: Newcastle–Ottawa Scale (NOS) Quality Assessment for Included 20 Studies.

| Study | Selection (0–4) | Comparability (0–2) | Outcome/Exposure (0–3) | Total Score (0–9) | Quality |
| --- | --- | --- | --- | --- | --- |
| Bodaghi, 2005 | 3 | 2 | 2 | 7 | High |
| Buyru, 2006 | 4 | 1 | 2 | 7 | High |
| Damin, 2007 | 3 | 2 | 3 | 8 | High |
| Laskar, 2015 | 3 | 2 | 3 | 8 | High |
| Lee, 2001 | 3 | 2 | 3 | 8 | High |
| Liu, 2011 | 4 | 2 | 3 | 9 | High |
| Picanco-Junior, 2014 | 3 | 2 | 3 | 8 | High |
| Salepci, 2009 | 3 | 2 | 2 | 7 | High |
| Tanzi, 2015 | 3 | 2 | 3 | 8 | High |
| Vuitton, 2017 | 4 | 2 | 3 | 9 | High |
| Yu, 2002 | 3 | 1 | 2 | 6 | Moderate |
| Zhang, 2012 | 3 | 1 | 3 | 7 | High |
| Tavakolian, 2020 | 3 | 2 | 2 | 7 | High |
| Galati, 2023 | 3 | 2 | 3 | 8 | High |
| Ambrosio, 2023 | 4 | 2 | 2 | 8 | High |
| Abedi Elkhichi, 2024 | 3 | 1 | 3 | 7 | High |
| Hsu, 2022 | 4 | 2 | 3 | 9 | High |
| Kadhem Mallakh, 2022 | 4 | 1 | 2 | 7 | High |
| Niya, 2022 | 4 | 1 | 2 | 7 | High |
| Pan, 2024 | 3 | 1 | 2 | 6 | Moderate |
