# Supplementary material for: Human papillomavirus (HPV) infection and prevalence of colorectal cancer: an updated systematic review and meta-analysis of global data
Source: Int J Surg. 2025 Sep 11;112(1):1815–25. doi: 10.1097/JS9.0000000000003426 (PMC12825822; doi:10.1097/JS9.0000000000003426)

Supplementary Figure 1. Subgroup analysis by sample type


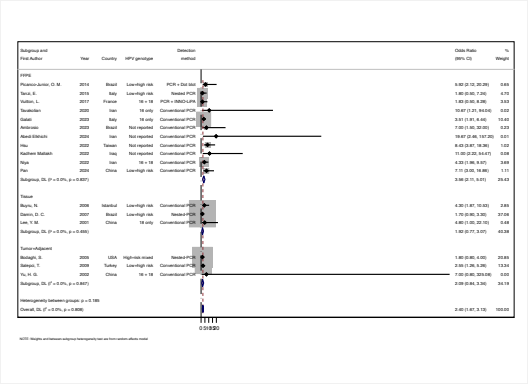


Supplementary Figure 2. Sub group by geographic region


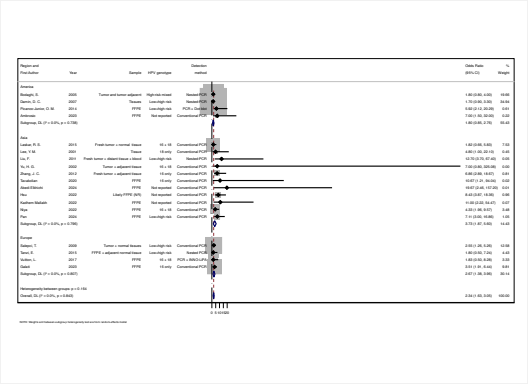


Supplementary Figure 3. Leave-One-Out Sensitivity Analysis


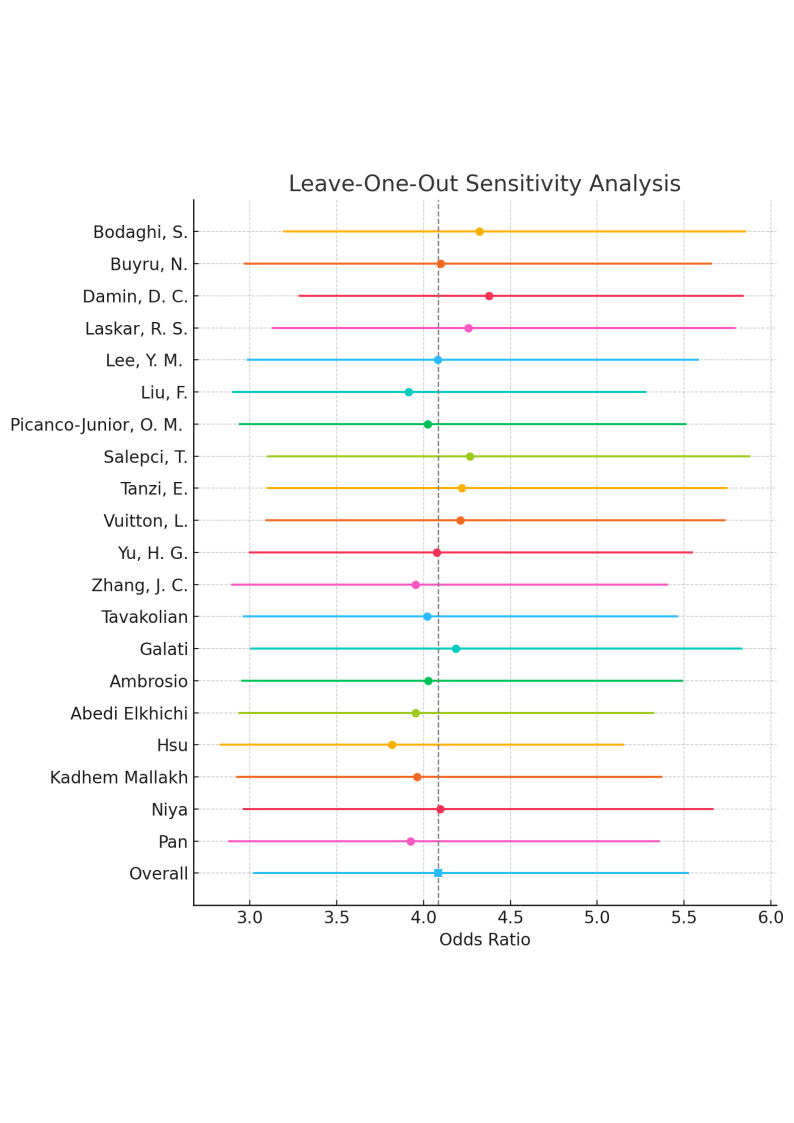


Supplementary Figure 4.Bubble Plot- HPV Genotype Group

| Supplementary Figure 5. Bubble Plot-Region |  |
| --- | --- |


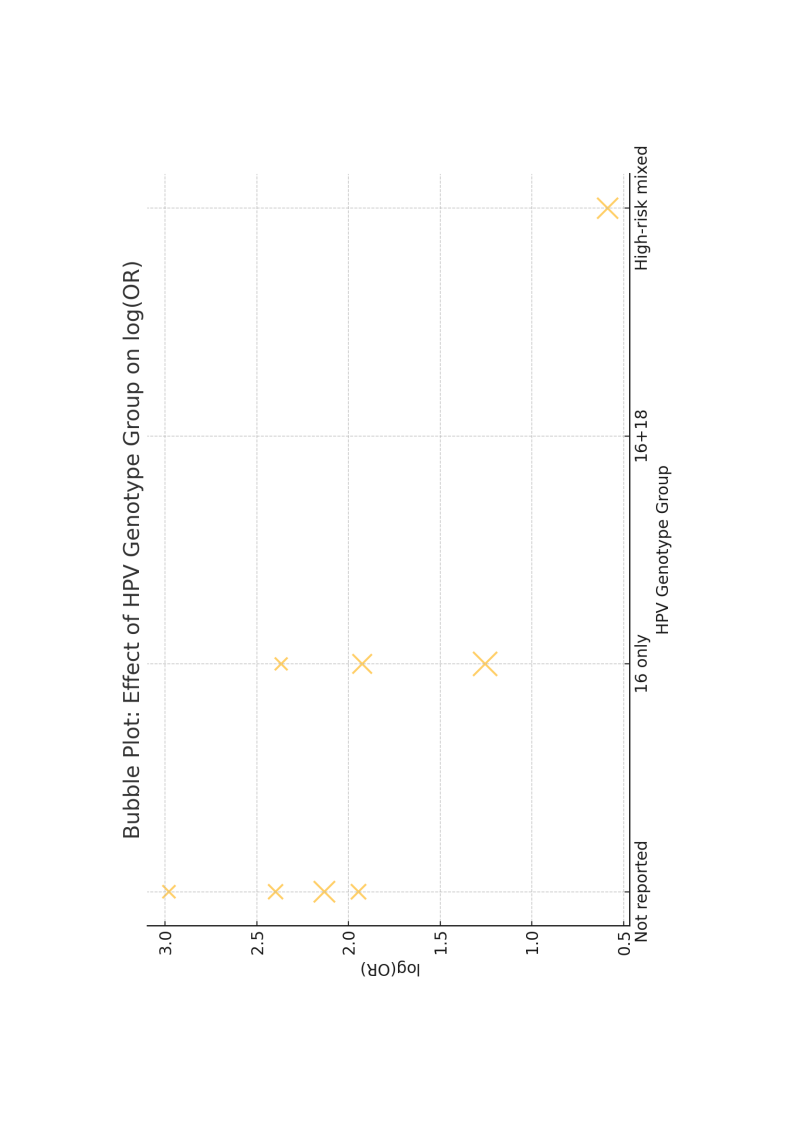


| Supplementary Figure 5. Bubble Plot-Region |  |
| --- | --- |


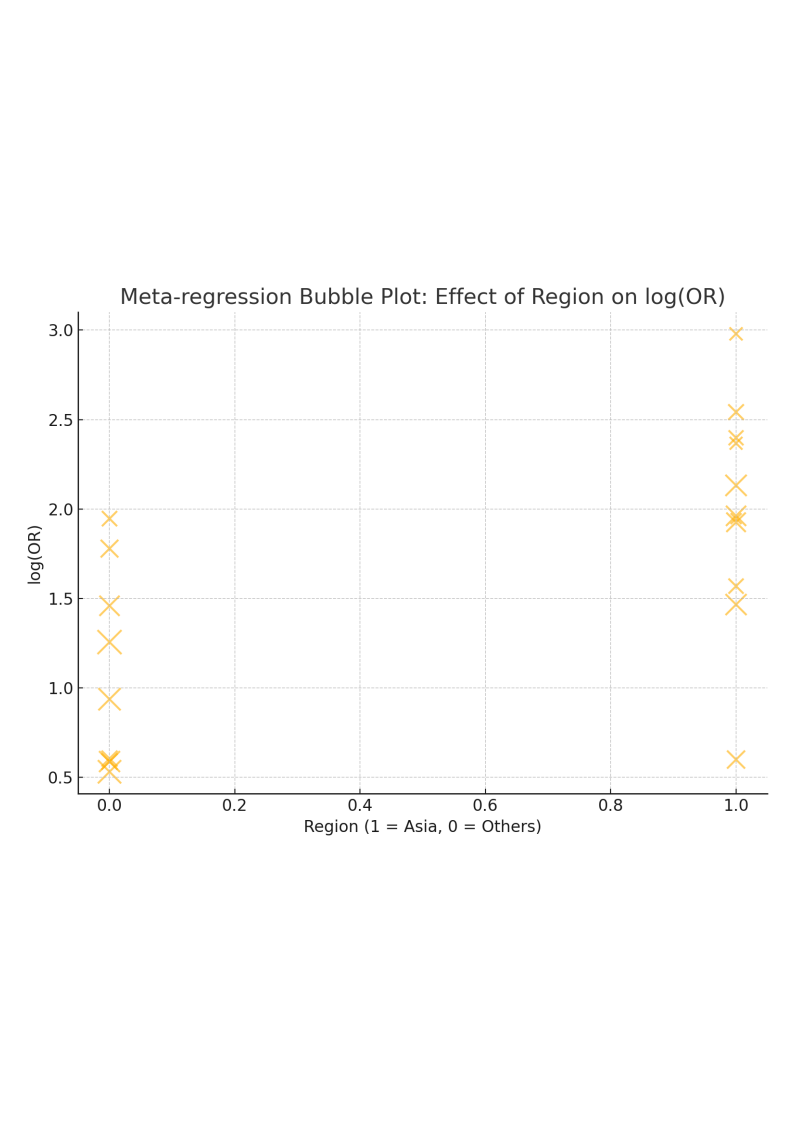


Supplementary Figure 6. Funnel plot assessing publication bias in studies reporting the association between HPV infection and colorectal cancer.


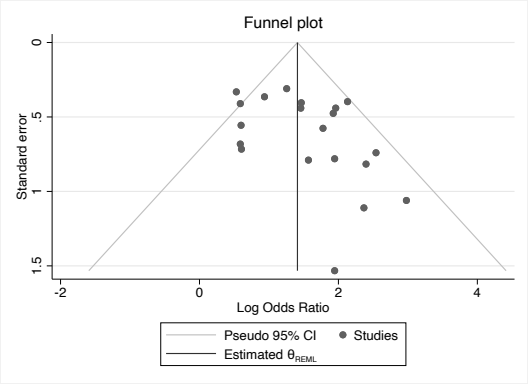

Supplement: Supplementary file 4 [file js9-112-1815-004.docx]
